# A Dataset for Fish Segmentation and Tracking in Underwater Videos

**DOI:** 10.1038/s41597-026-07786-z

**Published:** 2026-08-12

**Authors:** Josep Sanchez, Jose-Luis Lisani, Ignacio A. Catalan, Amaya Alvarez-Ellacuría

**Affiliations:** 1 Department of Mathematics and Informatics, University of the Balearic Islands, Palma de Mallorca, Spain; 2 Mediterranean Institute for Advanced Studies, Esportles, Spain

## Abstract

The automatic monitoring of fish in underwater imagery plays a key role in marine ecology, fisheries management, and environmental monitoring, yet progress is limited by the lack of large, high-quality fish-focused datasets. We present a new dataset of underwater videos of fish in natural habitats, annotated for pixel-level segmentation and multi-object tracking. The data was collected in the Balearic Sea, the western Mediterranean region surrounding the island of Mallorca (Spain), across diverse marine environments to capture variations in species, lighting, turbidity, and background complexity. Each video frame has been carefully annotated to ensure spatial and temporal consistency, yielding a challenging and comprehensive resource for developing and benchmarking underwater vision algorithms. To illustrate the dataset’s utility, we provide baseline tracking results obtained with Deep OC-SORT, which highlight both the dataset’s challenging nature and its potential for future method evaluation. In addition, we release an open-source, browser-based annotation tool integrating the Segment Anything Model (SAM2) and CUTIE for efficient semi-automatic segmentation and tracking. This tool facilitates high-quality annotations without specialized hardware, improving accessibility and reproducibility within the marine imaging community.

## Introduction

The study of marine organisms increasingly relies on video-based observation to monitor species behavior, population dynamics, and ecological interactions in their natural habitats^1–7^. Recent advances in computer vision and deep learning have made it possible to automatically detect, segment, and track marine animals in underwater imagery. However, progress in developing and evaluating such methods remains limited by the scarcity of annotated datasets suitable for segmentation and tracking. Existing underwater datasets often provide only image-level labels or coarse bounding boxes, such as those described in^8^ and^9^, with few offering temporally consistent instance annotations that enable multi-object tracking. Moreover, when such annotations exist, they are typically constrained to limited spatial or temporal scales. For example, the OzFish dataset^10,11^ contains a diverse collection of images but lacks temporal annotations. Other datasets may exhibit inconsistent labeling quality or be collected under controlled conditions that do not reflect the variability of natural marine environments. These limitations are particularly evident when compared with large-scale terrestrial benchmarks such as MOT20^12^ and YouTube-VIS^13,14^. As a result, there remains a pressing need for large, high-quality datasets that provide reliable segmentation and tracking annotations of fish in the wild.

To address these limitations, we present **SFISHTRACK**, a new dataset of **fish in the wild**, designed to support **segmentation** and object **tracking** tasks under diverse environmental conditions. The dataset includes high-resolution underwater videos with frame-level annotations, collected across multiple marine sites that capture a broad range of species, lighting, and turbidity conditions. We describe the data collection and annotation procedures, provide statistical analysis of the dataset, and compare its scope and characteristics with existing underwater datasets.

To facilitate future data annotation and extension, we also introduce an open-source, browser-based video annotation tool that integrates recent vision models to enable efficient and accurate segmentation and tracking directly within a web interface, without the need for dedicated hardware.

The remainder of this paper is organized as follows. First, we review related work on underwater datasets and annotation tools. Next, we describe the data collection process and annotation methodology, followed by a detailed presentation of the dataset structure. Finally, we provide a technical validation of the dataset and its annotations.

## Related works

### Object Tracking Datasets

The availability of high-quality datasets remains one of the primary constraints in advancing underwater object tracking research. Despite recent progress in underwater imaging technologies, publicly accessible datasets that are both large-scale and task-specific remain limited. Most existing collections are either too small to support data-hungry deep learning models or lack standardized metadata and annotation protocols necessary for reproducibility and cross-study comparison. These limitations are particularly acute in fish-tracking applications, where complex visual conditions and species variability demand rich, well-curated data resources.

When considering tracking datasets, most publicly available resources are designed for terrestrial (non-underwater) scenarios. These datasets typically focus on humans or commonly occurring objects such as animals, vehicles, or everyday items (e.g., cars and mobile phones). Among the most widely used benchmarks are human-centered datasets such as DanceTrack^15,16^ and MOT17 and MOT20^12,17–19^, which have been extensively adopted for evaluating multi-object tracking algorithms in structured environments.

In many of these datasets, annotations are provided in the form of bounding boxes that spatially localize objects within each frame. This representation is prevalent due to its relative simplicity and efficiency during the annotation process, making it suitable for large-scale dataset creation. However, bounding boxes offer only coarse localization and do not capture fine-grained object boundaries, which can limit performance in tasks requiring higher spatial precision.

Video instance segmentation datasets provide pixel-level annotations along with temporal consistency across frames. Notable examples include DAVIS 2017^20,21^ and YouTube-VIS^13,14^. These datasets enable more detailed analysis by jointly addressing segmentation and tracking, although they are still primarily focused on terrestrial scenes and general object categories.

### Underwater Datasets

Annotating underwater video sequences poses distinctive challenges compared to terrestrial imagery^22^. Water absorption and scattering significantly reduce contrast and color fidelity, while suspended particles and lighting inconsistencies introduce noise and spatial distortions. Moreover, the frequent occlusion, camouflage, and unpredictable motion patterns of fish further complicate the annotation process. Consequently, underwater datasets often exhibit inconsistencies in bounding box placement, temporal continuity, and annotation density. Addressing these challenges requires standardized collection methodologies, transparent metadata structures, and community-driven validation protocols elements that remain underdeveloped in current datasets.

In the context of underwater computer vision, most publicly available datasets are primarily designed for object detection rather than tracking. This reflects the inherent difficulty of acquiring temporally consistent annotations in underwater environments, where factors such as turbidity, lighting variability, and object occlusions significantly complicate the labeling process. Representative examples include HODOR^23^ and RMAS^24^, which provide annotated datasets for object detection with a particular emphasis on marine fauna, especially fish. These datasets have enabled the development of deep learning-based approaches for tasks such as species recognition, biodiversity assessment, and population counting, which are essential for ecological monitoring and fisheries management^25,26^.

Several datasets focus specifically on fish detection in underwater imagery. For instance, the OBSEA Fish Detection Training Dataset^27^ provides annotated images collected from underwater observatories, supporting research on automated fish detection under real-world marine conditions. Such datasets typically emphasize spatial localization and classification, often relying on bounding box annotations due to their relative ease of generation and scalability. The dataset originates from the OBSEA observatory, which has produced relevant underwater datasets, including the one mentioned above, as well as studies on marine ecosystem monitoring^28,29^.

Other underwater detection datasets extend beyond fish and include a broader range of object categories. The RUOD dataset^30^, for example, contains annotations for ten different classes of underwater objects, including marine organisms and man-made artifacts. This diversity supports the development of more general-purpose detection models capable of operating in complex underwater scenes.

More specialized datasets, such as OzFish^10,11^, offer higher-resolution imagery and improved annotation reliability. The dataset focuses explicitly on fish species and provides cleaner bounding boxes across controlled recording environments. However, its relatively homogeneous conditions, dominated by clear-water coastal scenes, restrict its ecological and visual diversity. As a result, it does not fully capture the variability found in natural underwater habitats, including turbidity, lighting gradients, and multi-species interactions.

**Fig. 1:**
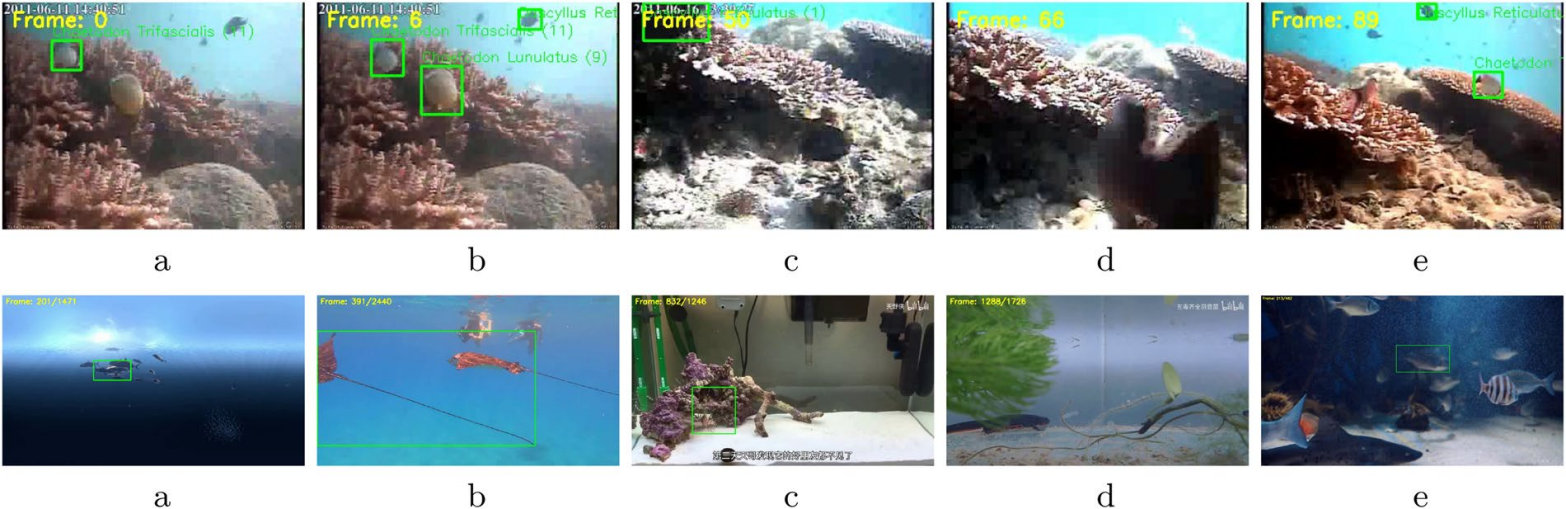
Examples of annotated frames from LifeCLEF^31^ (top) and WebUOT-1M^37^ (bottom). The displayed examples illustrate annotation inconsistencies observed in both datasets, including missing annotations in LifeCLEF and cases in WebUOT-1M where no object is annotated or a single bounding box encloses multiple objects.

Despite these advances, the focus on detection limits the applicability of these datasets for tasks requiring temporal consistency, such as multi-object tracking. Early efforts such as the LifeClef 2015 Fish Task dataset^31,32^ represented an important step toward the development of underwater tracking datasets. It provided multi-frame video sequences annotated with bounding boxes for fish detection. Although the dataset was originally designed for object detection, its video-sequence nature means it can also be used for tracking, provided that object identifiers are added to the annotations. However, its low spatial resolution, limited number of annotated sequences, and inconsistent labeling conventions (see Fig. 1 and the discussion in a later paragraph) restrict its compatibility with modern deep learning frameworks. Furthermore, metadata describing recording conditions and sensor characteristics were minimal, reducing reproducibility and limiting domain transferability.

Other underwater datasets that rely on video sequences as their primary data source include SeaCLEF^33^ and Fish4Knowledge^34^. These datasets can be regarded as early precursors to the development of underwater fish tracking benchmarks, as they provide valuable visual data captured in realistic marine environments. However, they still lack several key characteristics required to serve as primary sources for multi-object tracking (MOT) research. In particular, SeaCLEF does not provide temporal identity annotations, making it unsuitable for tracking tasks that require consistent object identities across frames. Although Fish4Knowledge includes video data and some trajectory-related information, its annotations are not standardized according to widely adopted MOT evaluation protocols. As a result, it cannot be directly employed as a benchmark for multi-object tracking without significant preprocessing or re-annotation efforts^35^.

Recent efforts have aimed to expand underwater tracking datasets in both scale and diversity. An important precursor is the UOT-100 dataset^36^, which provides 104 annotated video sequences and introduces a structured benchmark with varied visibility conditions and motion patterns. However, its relatively small scale limits its ability to capture the full variability of natural underwater environments.

Building on this, the WebUOT-1M dataset^37^ significantly increases scale, offering over one million annotated frames across diverse marine contexts. While this scale makes it valuable for training data-intensive models, the dataset aggregates heterogeneous sources with inconsistent annotation quality and a limited focus on ichthyofauna. Additionally, variations in file structures and annotation formats reduce metadata interoperability and complicate integration with other datasets.

A further limitation of both UOT-100 and WebUOT-1M is that, despite being tracking datasets, they are designed for single-object tracking (SOT). Although related to multi-object tracking (MOT), SOT differs substantially in its assumptions and evaluation protocols, which restricts the applicability of these datasets for tasks requiring simultaneous tracking of multiple fish.

Figure 1 presents several randomly selected sequences from the LifeCLEF 2015 dataset (top row) and the WebUOT-1M dataset (bottom row)^31,37^. For each dataset, frames from the selected sequences are displayed. In most LifeCLEF examples, missing annotations are evident. In some cases, this may reflect a deliberate decision by the annotators to exclude very small fish. However, if so, this criterion does not appear to have been applied consistently, since in some instances (e.g., images 1a and 1b) very similar fish are annotated in one frame but omitted in another frame from the same sequence. In WebUOT-1M, some frames contain no annotations (2 d), while in others the bounding box encompasses several objects (2b). Note that, in this dataset, at most one object is annotated per frame, since it is designed for single-object tracking.

Although such issues are not pervasive across all videos, they appear frequently enough to be noteworthy. These examples underscore the challenges of accurately annotating underwater video data, where visibility conditions and environmental factors are often suboptimal.

More recently, a dedicated underwater fish tracking dataset has been introduced named MFT25^35^. This dataset adopts a multi-object tracking framework and explicitly focuses on fish as the target objects, thereby satisfying many of the requirements for developing fish tracking solutions. However, its main limitation lies in the nature of the data acquisition: the sequences are captured in controlled or semi-controlled environments (e.g., fish farms or bounded settings), rather than in the wild. Consequently, models trained on this dataset may face challenges in generalizing to more complex and unconstrained natural marine environments.

In summary, although a variety of datasets exist for object detection, segmentation, and tracking, the development of robust underwater multi-object fish tracking systems remains constrained by the lack of large-scale, standardized, and ecologically representative benchmarks. Most available datasets either focus on terrestrial scenarios, prioritize detection over temporal consistency, or rely on single-object tracking formulations that are not directly applicable to multi-object settings. While recent underwater datasets such as UOT-100 and WebUOT-1M have expanded scale and introduced structured benchmarks, their primary limitation lies in their formulation as single-object tracking (SOT) datasets, which restricts their applicability to multi-object tracking scenarios despite their scale and diversity. Emerging multi-object fish tracking datasets address some of these gaps but are often restricted to controlled environments, limiting their ability to generalize to real-world marine conditions. Collectively, these challenges highlight the need for comprehensive, well-annotated, and diverse underwater datasets that support multi-object tracking in natural habitats, thereby enabling more reliable and transferable solutions for marine monitoring applications.

## Methods

### Data Acquisition and Provenance

**Fig. 2:**
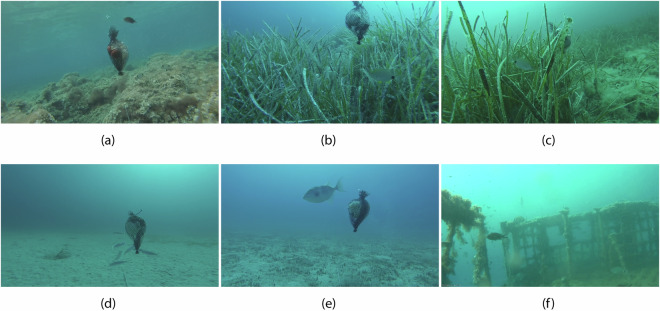
Examples of different marine environments, including seagrass meadows, rocky seabeds, wreck sites, and sandy plains. The six subimages illustrate representative visual variability across these habitats.

The dataset was compiled from underwater video recordings collected between 2021 and 2025 in the Balearic Sea, the Mediterranean subregion surrounding the island of Mallorca (Balearic Islands, Spain). The dataset was collected within the framework of long-term ecological monitoring programs coordinated by the Mediterranean Institute for Advanced Studies (IMEDEA). Examples of the environments represented in the dataset are shown in Fig. 2.

All videos were captured using stationary or diver-operated underwater video systems employing four different camera models, each enclosed in waterproof housings rated to the required operational depths. Some videos were captured on a wreck using fixed cameras, with Sony IPELA SNC-CH210 units recording the exterior and SAIS-IP-bullet cameras capturing the interior. Additional material was acquired using GoPro Hero 3 cameras deployed with baited setups, and GoPro Hero 7 cameras operated by scuba divers without bait. All systems used wide-angle lenses with automatic white-balance correction. Recordings were made at a resolution of 1920×1080 pixels (Full HD) and 30 frames per second (fps), and stored in MP4 (H.264) format. No artificial lighting was used in the deployments to minimize behavioral disturbance.

Cameras were deployed at depths ranging from 5 m to 40 m, covering diverse benthic and pelagic environments such as Posidonia oceanica seagrass meadows, rocky seabeds, wreck sites, and sandy plains. Each deployment varied in duration. Recordings from the fixed cameras were captured continuously, and segments were later selected for inclusion based on predefined practical criteria rather than fully automatic objective metrics. In particular, we aimed to ensure diversity and usability by limiting the number of selected segments from the same source recording and favoring segments with visible fish in most frames, moderate fish density, acceptable lighting and noise conditions, and durations between 15 and 25 seconds. This process involved a degree of subjectivity, but was designed to obtain a representative sample of recording conditions and fish activity patterns. The other camera systems were operated in separate sessions, each with its own deployment time.

For every deployment, contextual metadata were recorded and synchronized with the video timestamps. These include: Date and local time of recording, depth (m), camera model and approximate location, to protect sensitive marine sites. Environmental data were logged using onboard sensors and dive logs, and all metadata were formatted according to a structured schema (JSON).

**Fig. 3:**
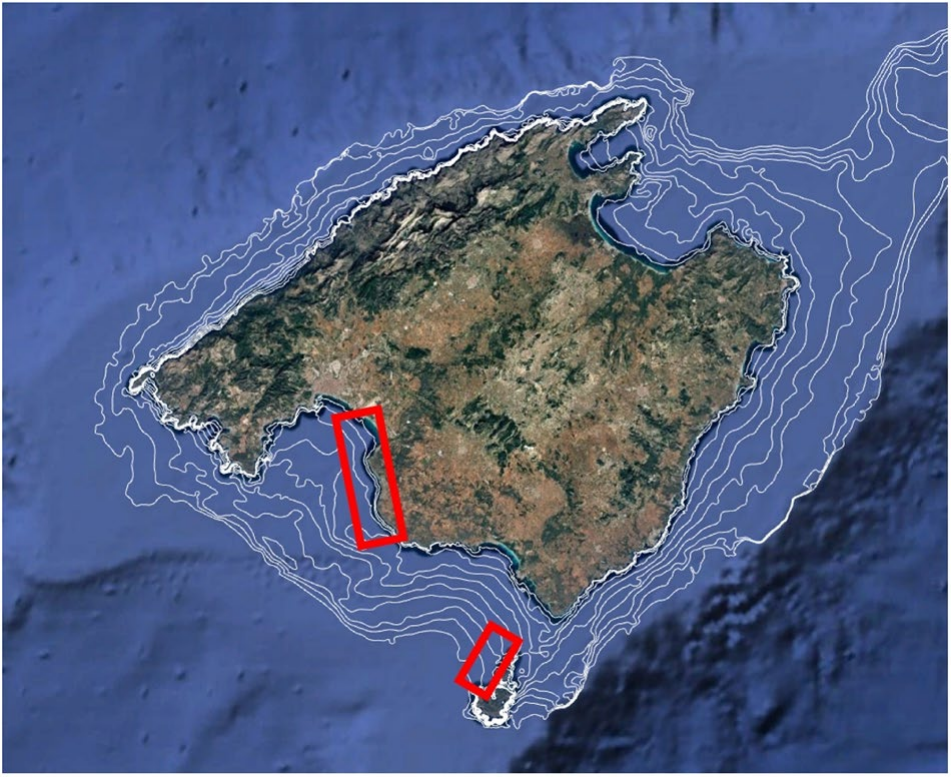
Map with the location of the cameras around Mallorca.

Figure 3 illustrates the approximate locations of all camera deployments around the island of Mallorca, where the underwater videos in the SFISHTRACK dataset were recorded. The map displays the spatial distribution of the sampling sites along the coastline, highlighting the geographic diversity of the monitored areas while omitting precise coordinates to protect sensitive marine habitats. The two main areas were the coastline around Palma, the capital city of Mallorca, and the waters near Cabrera, the island south of Mallorca.

**Fig. 4:**
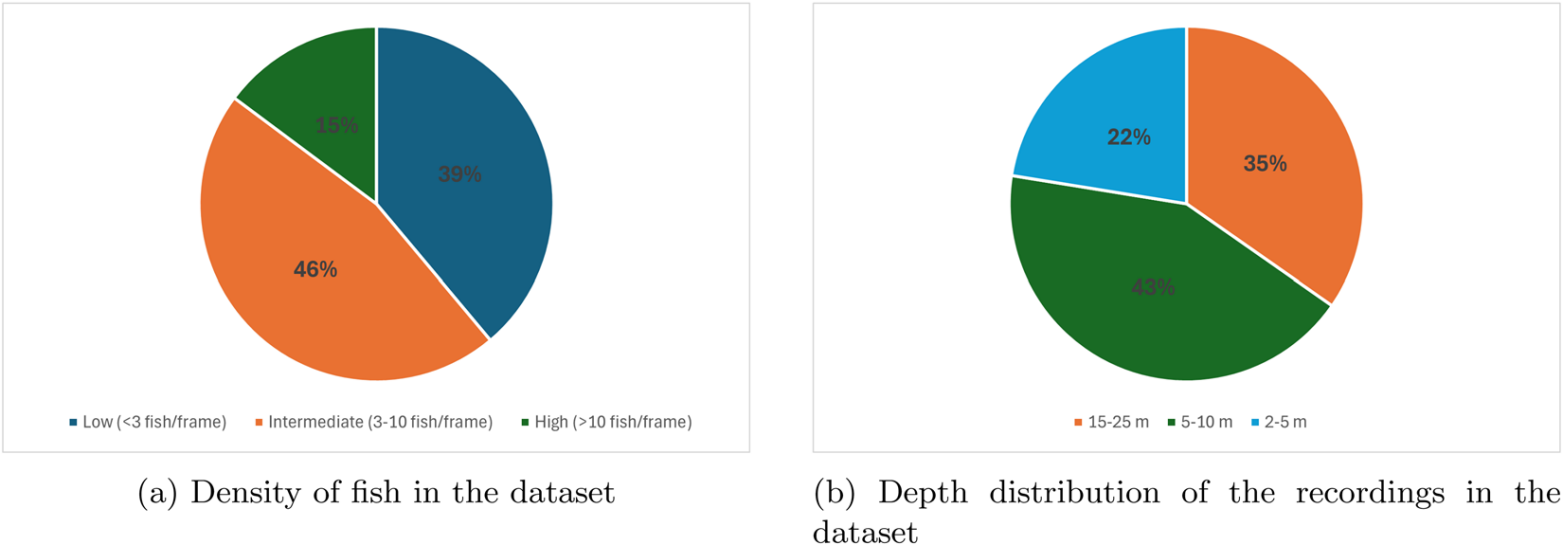
Dataset statistics: fish density and depth distribution.

Figure 4a summarizes the ecological diversity of the dataset by depicting fish density per frame, thereby reflecting the range of visual complexity represented in the data.

Figure 4b illustrates the depth distribution of the video recordings included in the dataset. Most sequences were captured at shallow to mid-range depths, reflecting the operational range of the deployed camera systems. The presence of recordings across the full depth span from 5 m to 25 m introduces meaningful variability in illumination, color attenuation, and background structure. This depth diversity contributes to the visual heterogeneity of the dataset and increases its relevance for evaluating tracking and segmentation methods under different underwater imaging conditions.

**Fig. 5:**
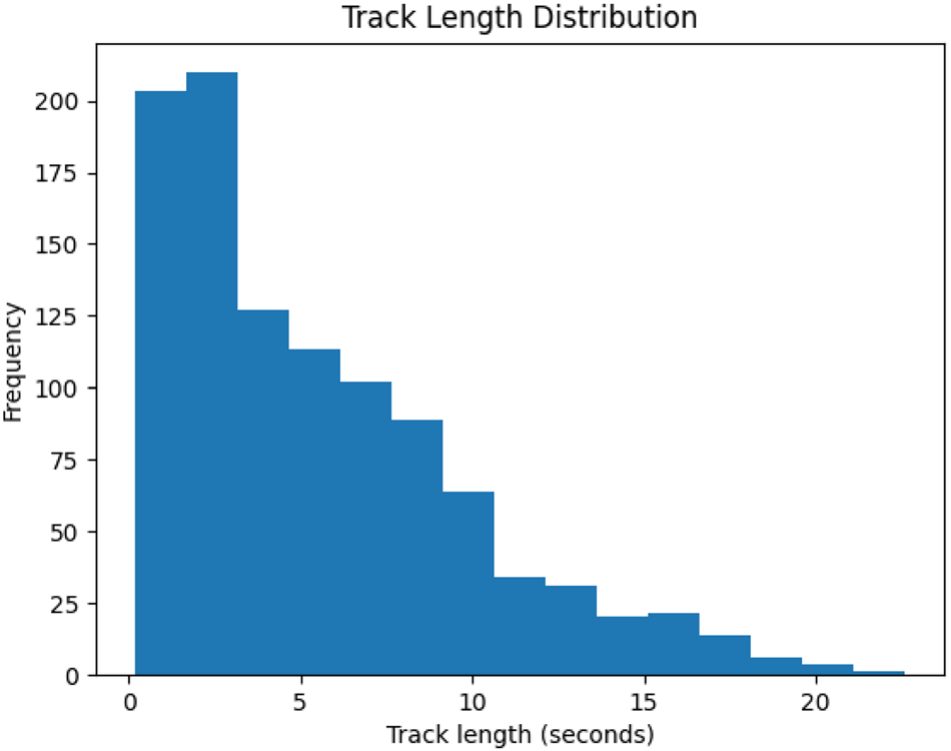
Dataset statistics: track length distribution.

These recordings have an average duration of 15.13 seconds and contain about 446 frames each, contributing to a cumulative total of 24,084 frames across all entries. On average, each video contains 17.57 distinct fish identities, and the mean number of annotated fish per frame is 6.26. The mean track length is 5.2 seconds, indicating that annotated identities are typically observed over temporally meaningful intervals. The full distribution of track lengths is shown in Fig. 5.

The complete dataset, including raw videos, annotation masks, and structured metadata, is hosted on Zenodo, with the link provided in our GitHub repository, which also contains the code and documentation. All files are version-controlled and distributed under a CC BY 4.0 license, ensuring long-term accessibility and reproducibility.

### Data Processing and Annotation Pipeline

Each video was processed using a custom web-based annotation interface that integrates two open-source segmentation models: the Segment Anything Model (SAM2)^38^ for generating initial object masks and Cutie^39^ for temporal mask propagation across frames. These models were used as semi-automatic annotation tools and incorporated into the annotation workflow developed for this study (see Section 3.4).

**Fig. 6:**
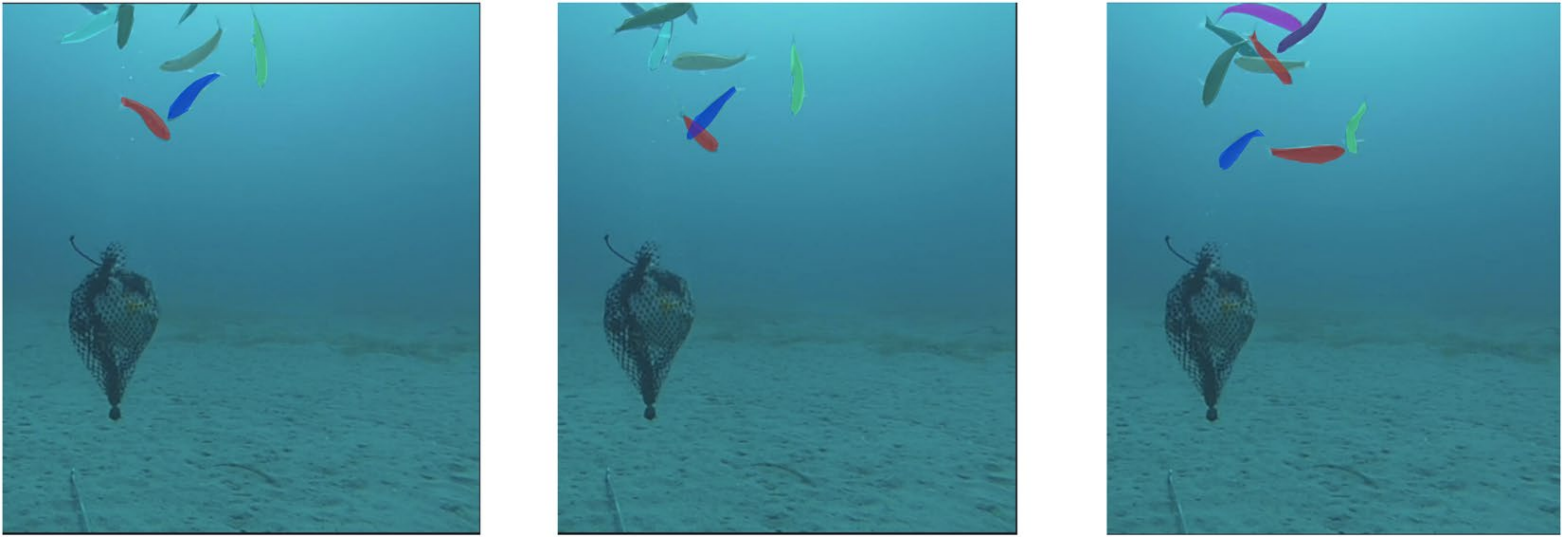
Example of three frames of annotated data in the SFISHTRACK dataset. The color of each mask denotes the identifier of the fish.

The annotation pipeline proceeded as follows. First, SAM2 was applied to generate instance-level segmentation masks for fish in each video using keypoint prompting, in which annotators provided point annotations on the target instances to guide mask generation. These initial masks, along with their associated object identifiers, were subsequently propagated through the sequence using Cutie to enforce temporal consistency across frames. The propagated masks were treated as preliminary annotations and were manually reviewed and refined using correction tools provided by the annotation interface. During this review, annotators corrected missing or imprecise regions, resolved overlapping masks, and addressed identity inconsistencies arising from long occlusions or partial visibility, which could otherwise result in identifier swaps between closely spaced individuals. Fish entering or leaving the field of view were assigned new identities, as continuous tracking outside the visible area was not possible. An example of annotated data is shown in Fig. 6.

Model parameters, such as the propagation window, were empirically adjusted to improve mask stability during rapid motion and occlusion events. The annotation interface, together with the data processing scripts, configuration files, and usage instructions, is publicly available in the accompanying GitHub repository, ensuring full reproducibility of the annotation pipeline.

As a general annotation guideline, fish instances that were too small to exhibit distinguishable visual characteristics were excluded from the labeling process. Specifically, a threshold of 25×25 pixels was adopted to determine whether a fish could be reliably annotated. Objects below this size often lack sufficient detail for consistent identification and precise segmentation, increasing the likelihood of annotation errors and inter-annotator variability. Moreover, prior work^40^ has shown that the inclusion of very small objects can negatively impact model performance, particularly in detection and tracking tasks where noise and ambiguity are amplified. Considering both the reduced annotation reliability and the potential adverse effects on model training, excluding such small instances represents a principled and practical design choice.

### Annotation Validation and Quality Control

Two trained annotators conducted the labeling over a period of six months. Annotations were stored in COCO JSON format to ensure interoperability with standard computer vision frameworks. To ensure labeling accuracy, a multi-stage validation process was applied:

- Automated integrity checks of the annotation files.
- Detection of missing masks and duplicate identifiers.
- Cross-review audits, in which each annotator reviewed 10% of the other annotator’s labels to assess identifier consistency.
- Quantitative validation, in which a randomly selected subset of 500 frames was independently re-annotated to estimate inter-annotator agreement.

### Computational Environment and Reproducibility

We decided to design our own annotation interface to ensure consistency of the annotations and to improve the speed of the task. The initial implementation of our annotation interface was a desktop application^41^, whereas the version used in this work has been redesigned as a web-based system. The use of a web-based platform significantly improves accessibility and scalability. Computationally intensive tasks run on a centralized server, enabling remote annotation on low-resource devices through a web browser. The system supports concurrent users, facilitating collaborative workflows critical for large-scale projects. The user interface was also designed for clarity, responsiveness, and ease of use. The system is well-suited for high-precision, efficient annotation.

**Fig. 7:**
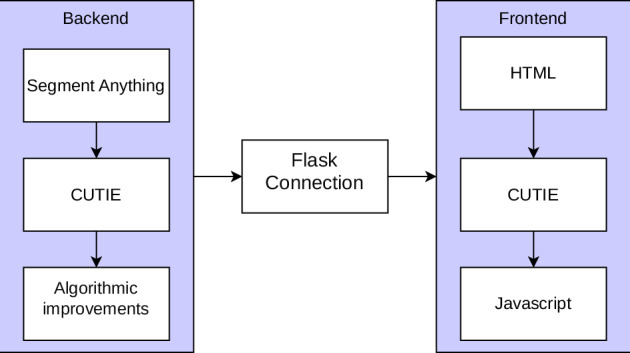
System Architecture of the annotation tool.

The system uses Flask to integrate Python-based inference with a JavaScript frontend, streamlining user interaction and minimizing configuration overhead. Two deep learning models are deployed server-side: the Segment Anything Model (SAM2) for prompt-based object segmentation, and Cutie for propagating and refining masks across video frames. SAM2 supports fast, point-based annotations, while Cutie enhances temporal consistency. Users can refine masks manually and correct propagation errors as needed, maintaining both speed and accuracy in annotation. Figure 7 provides a visual representation of the web application’s system architecture and the pipeline described above.

The annotation interface was run on Linux systems equipped with NVIDIA GPUs to accelerate segmentation and mask propagation. The workflows were tested on two configurations: (1) a single NVIDIA RTX 3060 (12 GB VRAM) and (2) dual NVIDIA RTX A6000 (48 GB VRAM each). Both environments successfully executed the pipeline within practical runtimes. Environment specifications are defined in an accompanying environment.yml available in the project repository.

## Data records

**Fig. 8:**
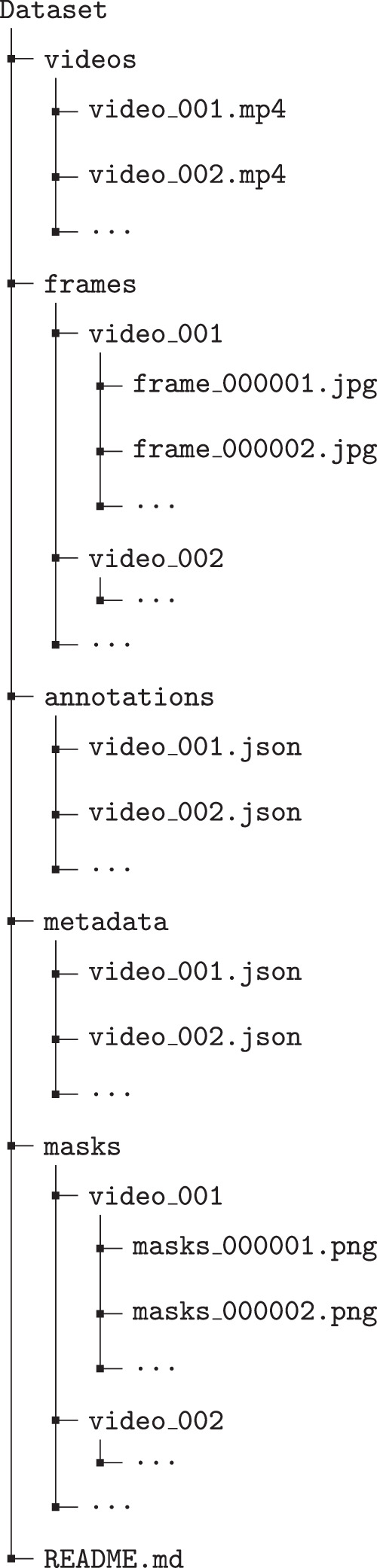
Directory tree of the dataset.

The dataset can be obtained from a link in our GitHub repository https://github.com/JosepSanchezCano/SFISHTRACK/ and from the Zenodo repository^42^. It has a total size of approximately 20 GB and contains 24,084 frames corresponding to 54 different videos, all of which are fully annotated with fish segmentation masks. All frames are provided at a resolution of 1920×1080 pixels. The original videos were rescaled to this standardized format to ensure consistency across the dataset, while minimizing information loss for potential use with super-resolution algorithms. In addition to the original frames and JSON annotation files, the dataset also includes a masks folder containing one mask image per frame for each sequence. These mask images provide a direct pixel-level visualization of the annotations and facilitate inspection, validation, and practical use of the dataset. The directory structure of the dataset is shown in Fig. 8.

The dataset annotations follow the standard COCO JSON format. In this section, we describe the key annotation fields and the information required to fully understand and use the dataset. For each video, a single JSON file is provided containing the annotations and associated metadata. Each JSON file includes the following key-value pairs:

1. Images: This key represents information associated with each video frame.
  - width: Image width, in pixels.
  - height: Image height, in pixels.
  - id: Image identifier, used for internal reference within the annotation file.
  - file_name: Name of the image file associated with the identifier.
2. Categories: This key defines the object classes included in the dataset. In the current version, all annotated instances belong to a single class (*fish*), and no species-level labels are provided. Future extensions of the dataset may include individual fish species as separate classes.
  - id: Class identifier.
  - name: Class name.
  - supercategory: Name of a higher-level category grouping related classes.
3. Annotations: This key contains the annotation data associated with each object instance.
  - area: Number of pixels in the segmented region.
  - bbox: Bounding box enclosing the segmentation mask.
  - category_id: Identifier of the object class.
  - frame_number: Index of the video frame associated with the annotation.
  - id: Annotation identifier.
  - image_id: Identifier of the corresponding image.
  - iscrowd: Flag indicating whether the annotation represents multiple objects.
  - segmentation: Polygon coordinates defining the segmentation mask.
4. Recording data: This key stores metadata associated with the video acquisition.
  - date: Date of the recording.
  - local_time: Time of the recording.
  - camera_model: Model of the camera used to capture the video.
  - depth: Recording depth.
  - location: Approximate geographic location of the camera.
  - baited: Boolean field indicating whether the recording was obtained under baited or unbaited conditions.

## Technical Validation

The quality and reliability of the annotated data were evaluated through both quantitative and qualitative assessments. Validation focused on two main aspects: the spatial precision of segmentation masks relative to fish boundaries, and the temporal continuity of fish identifiers across frames, ensuring consistent tracking throughout video sequences. Because all fish were annotated as a single class and no species-level labels were included, the evaluation focused exclusively on spatial and temporal annotation quality.

### Manual verification and error correction

Following automated mask generation, all annotated sequences underwent manual validation by trained annotators. Each sequence was reviewed frame by frame to ensure: masks accurately delineated fish contours, no fish regions were omitted (e.g., fins, tails), and identity assignments remained consistent across occlusions, crossings, and reappearances.

An important challenge in annotating underwater fish imagery is deciding how to handle very small individuals that appear predominantly in the background. To address this, we established a size-based exclusion criterion. Specifically, fish measuring less than 25×25 pixels, or whose fins and tail were not clearly distinguishable without zooming, were not annotated. This decision was made to reduce ambiguity, as such small fish are prone to being visually confused with background noise, particulate matter, or other artifacts, thereby introducing inconsistency and reducing the reliability of both detection and tracking tasks.

Common annotation errors included over-segmentation (mask leakage), partial omission of extremities, and temporary identifier mismatches. These errors were systematically documented and corrected using an iterative feedback process. Specifically, identified discrepancies were used to refine mask propagation parameters and improve initialization procedures in subsequent annotation batches.

This iterative correction loop resulted in measurable improvements: in a subset of 1,000 frames re-annotated after the third iteration, the mean Intersection over Union (IoU) between automated and verified masks increased from 0.84 to 0.92, while identifier mismatches decreased by 37%.

To quantify annotation reliability, we computed inter-annotator agreement on a randomly selected subset of 500 frames labeled independently by two annotators. For this experiment, the annotators worked independently and did not share the same SAM2-generated initial masks. The mean IoU across corresponding masks was 0.89, indicating high spatial consistency. Temporal consistency was evaluated by comparing the proportion of uninterrupted identity sequences across frames, yielding a continuity greater than 90%.

### Dataset comparison and benchmarking

Table 1 presents a comparative overview of datasets across three categories: non-underwater tracking and segmentation datasets, underwater detection datasets, and underwater tracking datasets. This comparison highlights the differences in scale, annotation type, and domain-specific challenges, providing context for the positioning of the proposed dataset.

**Table 1:** Comparison of datasets across domains and tasks. ^1^For underwater detection datasets, only the number of frames is reported, as they do not include tracking annotations and may not originate from video sequences, with the exception of LifeClef 2015 and HODOR.

Dataset	Videos	Frames	Duration (hr)	Mean (min)	Segm.
**Non-underwater tracking and segmentation datasets**					
MOT17	42	17,757	0.16-0.35	0.23-0.5	×
MOT20	8	13,375	0.15	66.87	×
DanceTrack	100	105,000	1.46	0.88	×
YouTube-VIS	2,883	80,000	0.75	0.015	✓
DAVIS 2017	150	10459	0.1	0.08	✓
**Underwater detection datasets**^1^					
HODOR	—	28.8 M	400	—	×
RMAS	—	3014	—	—	×
RUOD	—	14000	—	—	×
OBSEA	—	4120	—	—	×
OzFish	—	1800	—	—	×
LifeClef 2015	20	20 K	0.20	0.60	×
**Underwater tracking datasets**					
Fish4Knowdledge	+4000	700 K	3000	—	×
UOT100	104	74 K	0.68	0.39	×
WebUOT-1M	1,500	1.1 M	10.5	0.42	×
MFT25	15	48 K	0.44	1.7	×
SFISHTRACK	54	24.1 K	0.35	0.33	✓

Non-underwater datasets such as MOT17, MOT20, DanceTrack, DAVIS 2017, and YouTube-VIS are generally well-established benchmarks with relatively large-scale annotations and standardized evaluation protocols. These datasets benefit from controlled imaging conditions, higher visibility, and more consistent object appearances. As a result, they often provide reliable annotations, including bounding boxes or pixel-level segmentation masks, and support both tracking and segmentation tasks. In particular, datasets like DAVIS 2017 and YouTube-VIS incorporate high-quality segmentation annotations, enabling more precise spatial understanding compared to detection-based approaches.

In contrast, underwater detection datasets, including HODOR, RMAS, RUOD, OBSEA, and OzFish, primarily focus on object localization rather than temporal tracking. These datasets vary significantly in scale, ranging from a few thousand images to tens of millions of frames, as in the case of HODOR. However, they typically lack temporal annotations and consistent identity labeling across frames, limiting their applicability to tracking tasks. Furthermore, many of these datasets are not derived from continuous video sequences, which further constrains their use in modeling temporal dynamics. Despite these limitations, they remain valuable for tasks such as species detection, biodiversity monitoring, and object classification in challenging underwater conditions.

Underwater tracking datasets, including LifeClef 2015, UOT100, WebUOT-1M, MFT25, and Fish4Knowledge, represent important steps toward incorporating temporal information into underwater visual analysis. Nevertheless, they still involve trade-offs in scale, annotation quality, task design, and domain specificity. Notably, UOT100 and WebUOT-1M are single-object tracking datasets, which makes them unsuitable for training or evaluating methods intended to track multiple fish simultaneously. LifeClef 2015 includes temporally structured fish sequences, but its low spatial resolution and annotation inconsistencies limit its usefulness for modern deep learning pipelines. Fish4Knowledge provides many frames, yet it lacks segmentation annotations and detailed metadata. By contrast, SFISHTRACK offers high-quality pixel-level segmentation, consistent identity tracking, and a dedicated focus on fish in diverse natural environments, making it a stronger benchmark for underwater multi-object tracking.

To assess dataset usability and challenge level, we benchmarked SFISHTRACK using Deep OC-SORT^43^, a multi-object tracking framework that combines motion cues with appearance-based association for temporal linking. Performance was evaluated using the standard MOT metrics MOTA, MOTP, IDF1^44^, and HOTA^45^. All metrics are reported as percentages, and identical evaluation scripts were used across all datasets to ensure consistency.

The benchmark was designed as a cross-dataset comparison under a fixed tracking pipeline. No additional training or fine-tuning was performed on any of the evaluated datasets. Instead, we used Ultralytics YOLOv8m, initialized with the default pretrained medium-sized weights, to generate object detections for each frame, and these detections were provided directly as input to Deep OC-SORT. The default Deep OC-SORT configuration provided by the original authors was used throughout all experiments. All available annotated sequences in each dataset were included in the evaluation.

Since Deep OC-SORT operates on bounding-box detections, all evaluations were conducted in the bounding-box domain. For datasets annotated with segmentation masks, each annotated instance was converted to its enclosing bounding box before evaluation. This allowed all datasets to be assessed under the same detection-and-tracking protocol.

It is important to note that UOT100 and WebUOT-1M are single-object tracking (SOT) datasets. In these datasets, at most one object is annotated per frame, which differs substantially from the annotation structure of conventional multi-object tracking benchmarks. Nevertheless, we included them in the benchmark because of the limited availability of fish-focused tracking datasets. Their results should therefore be interpreted with caution: when evaluated with MOT metrics, any additional detections are counted as false positives, even when they correspond to valid fish in the scene. As a result, metrics such as MOTA may be artificially lowered and are not directly comparable to those obtained on genuine MOT datasets.

To extend the benchmarking analysis, we augmented the LifeCLEF 2015 dataset with consistent object identifiers, thereby enabling its use for multi-object tracking evaluation. Because the original annotations do not provide persistent identities across frames, we introduced temporal identity labels to ensure compatibility with standard MOT metrics. To this end, we first employed a tracking-by-detection approach to automatically assign identifiers, which were subsequently manually reviewed and revised to improve consistency.

Table 2 provides an overview of how the tracker behaves across a diverse set of underwater datasets, offering a comparative perspective on its strengths and limitations under different annotation protocols and scene conditions. Rather than focusing only on absolute values, the table highlights how performance varies depending on factors such as object density, annotation strategy, and visual complexity. Datasets such as SFISHTRACK, together with LifeCLEF 2015 after the addition of temporal identities, are closer to conventional multi-object tracking settings, and therefore the reported metrics provide a more direct joint assessment of detection, localization, and identity preservation. The MFT25 result is included as an additional literature-based reference point and should be interpreted accordingly.

**Table 2:** Performance of Deep OC-SORT on underwater tracking datasets.

Dataset	MOTA (%)↑	MOTP (%)↑	HOTA (%)↑	IDF1 (%)↑
LifeClef 2015	44.21	45.34	24.99	44.16
UOT100	19.89	58.52	21.47	51.01
WebUOT-1M	14.52	60.12	20.32	48.79
MFT25^2^	46.72	—	24.85	34.18
SFISHTRACK (ours)	41.00	42.21	26.31	32.92

^2^Values for MFT25 were extracted from^37^, Table 2, and are included for contextual comparison only; they were not recomputed in this work.

By contrast, the results obtained on UOT100 and WebUOT-1M should be interpreted with caution, since their single-object annotation protocol penalizes additional detections as false positives. Consequently, metrics such as MOTA are strongly influenced by the dataset design and are not directly comparable to those obtained on conventional MOT datasets. Overall, the table is intended to help contextualize tracker performance across datasets with different characteristics, rather than to support a purely rank-based comparison^46,47^.

## Data Availability

The dataset is publicly available for anonymous download through the following GitHub repository: https://github.com/JosepSanchezCano/SFISHTRACK. It has not been partitioned into predefined subsets, as its wide range of potential applications requires task-specific dataset configurations. The dataset is also available through Zenodo; a link to the corresponding Zenodo repository is provided in the aforementioned GitHub repository and can additionally be found in^48^.

## Code availability

The dataset annotations are provided in the COCO JSON format, ensuring seamless integration without the need for additional code. The video annotation tool can be found at https://github.com/JosepSanchezCano/UVAT-web-app. The GitHub provides the code and the installation instructions. The weights for SAM2 are downloaded upon first execution of the application and Cutie weights can be found at https://github.com/hkchengrex/Cutie and be copied into the “weights” folder.

## References

[1] Aguzzi, J. *et al*. The potential of video imagery from worldwide cabled observatory networks to provide information supporting fish-stock and biodiversity assessment. *ICES Journal of Marine Science* **77**(7-8), 2396–2410, 10.1093/icesjms/fsaa169 (2020).

[2] Condal, F. *et al* Cabled video-observatory monitoring of seasonal rhythms in a mediterranean coastal fish community. Hal preprint hal-03013945, (2020).

[3] Glaviano, F. *et al*. Management and sustainable exploitation of marine environments through smart monitoring and automation. *Journal of Marine Science and Engineering* **10**(2), 297, 10.3390/jmse10020297 (2022).

[4] Nalmpanti, M. *et al*. Monitoring marine fishes using underwater video techniques in the mediterranean sea. *Reviews in Fish Biology and Fisheries* **33**, 1291–1310, 10.1007/s11160-023-09799-y (2023).

[5] Buscaino, G. *et al*. Integrated video and acoustic monitoring of coastal fish biodiversity using a cabled observatory. *Ecological Indicators* **178**, 114025, 10.1016/j.ecolind.2025.114025 (2025).

[6] Domingo, F. C. Urban marine biodiversity monitoring by environmental science students. *International Journal of Marine Science* **14**(6), 341–349, 10.5376/ijms.2024.14.0038 (2024).

[7] Gröger, J. P. *et al*. Development and operation of a novel non-invasive opto-acoustic underwater fish observatory in kiel bight, southwestern baltic sea. *Frontiers in Marine Science* **11**, 1425259, 10.3389/fmars.2024.1425259 (2024).

[8] Saleh, A. *et al*. A realistic fish-habitat dataset to evaluate algorithms for underwater visual analysis. *Scientific Reports* **10**(1), 14671 (2020). 32887922 10.1038/s41598-020-71639-x PMC7473859

[9] Ditria, E. M. *et al*. Automating the analysis of fish abundance using object detection: Optimising out-of-sample generalisation requires more than big data. *Frontiers in Marine Science* **7**, 561893 (2020).

[10] Watson, M. Ozfish dataset: machine learning dataset for baited remote underwater video stations. CSIRO Data Collection (2020).

[11] Watson, M. *OzFish Dataset: Machine Learning Dataset for Baited Remote Underwater Video Stations*, CSIRO Data Collection, Available at: https://researchdata.edu.au/ozfish-dataset-machine-learning/1333757, accessed June 2026.

[12] Dendorfer, P. *et al*. Mot20: A benchmark for multi-object tracking in crowded scenes. arXiv preprint arXiv:2003.09003, 10.48550/arXiv.2003.09003 (2020).

[13] Yang, L., Fan, Y. & Xu, N. Video instance segmentation. In *Proceedings of the IEEE/CVF international conference on computer vision*, pages 5188-5197, 10.1109/ICCV.2019.00529 (2019).

[14] Yang, L., Fan, Y. & Xu, N. *YouTube-VIS: Video Instance Segmentation Dataset*, Available at: https://youtube-vos.org/dataset/vis/, accessed June 2026.

[15] Sun, P. *et al* Dancetrack: Multi-object tracking in uniform appearance and diverse motion. In *Proceedings of the IEEE/CVF conference on computer vision and pattern recognition*, pages 20993-21002, 10.1109/CVPR52688.2022.02032 (2022).

[16] DanceTrack Team, *DanceTrack Dataset and Benchmark*, Available at: https://dancetrack.github.io/, accessed June 2026.

[17] MOTChallenge Team, *MOT20: Multiple Object Tracking Benchmark Dataset*, MOTChallenge, Available at: https://motchallenge.net/data/MOT20/, accessed June 2026.

[18] Dendorfer, P. *et al*. Motchallenge: A benchmark for single-camera multiple target tracking. *International Journal of Computer Vision* **129**(4), 845–881, 10.1007/s11263-020-01393-0 (2021).

[19] MOTChallenge Team, *MOT17: Multiple Object Tracking Benchmark Dataset*, MOTChallenge, Available at: https://motchallenge.net/data/MOT17/, accessed June 2026.

[20] Pont-Tuset, J. *et al*. The 2017 davis challenge on video object segmentation. arXiv preprint arXiv:1704.00675, 10.48550/arXiv.1704.00675 (2018).

[21] DAVIS Team, *DAVIS: Densely Annotated Video Segmentation Dataset*, Available at: https://davischallenge.org/, accessed June 2026.

[22] Cutter, G., Stierhoff, K. & Zeng, J. Automated Detection of Rockfish in Unconstrained Underwater Videos Using Haar Cascades and a New Image Dataset: Labeled Fishes in the Wild. in *Proceedings of the 2015 IEEE Winter Conference on Applications of Computer Vision Workshops (WACVW)*, Waikoloa, HI, USA, pp. 57–62, 10.1109/WACVW.2015.11 (2015).

[23] Wilts, T. *et al*. Hodor: Hydroacoustic and optical dataset for oceanic research. *IEEE Data Descriptions* **2**, 262–270, 10.1594/PANGAEA.980000 (2025).

[24] Fu, Z. *et al*. Masnet: A robust deep marine animal segmentation network. *IEEE Journal of Oceanic Engineering* **49**(3), 1104–1115, 10.1109/JOE.2023.3252760 (2023).

[25] Australian Research Data Commons (ARDC), *FishID: A Robust and Intuitive AI System for Researchers to Annotate Imagery and Train and Evaluate Deep Learning Models*, [Online]. https://ardc.edu.au/project/fishid/. Accessed: Jun. 4, 2026 (2022).

[26] Villon, S. *et al*. A deep learning method for accurate and fast identification of coral reef fishes in underwater images. *Ecological Informatics* **48**, 238–244, 10.1016/j.ecoinf.2018.09.007 (2018).

[27] Banos Castello, P. *et al*.Underwater images from obsea fish detection training dataset (yolo). *Zenodo*10.5281/zenodo.14888440 (2025).

[28] Francescangeli, M., Río, J. D., Nogueras, M., Chatzievangelou, D. & Aguzzi, J. Implementation of the OBSEA coastal seafloor observatory to derive ecological indicators, in *Proceedings of the VII International Symposium on Marine Sciences (ISMS 2020)*, Barcelona, Spain (2020).

[29] Condal, F. *et al*. Seasonal rhythm in a Mediterranean coastal fish community as monitored by a cabled observatory. *Marine Biology* **159**(12), 2809–2817, 10.1007/s00227-012-2041-3 (2012).

[30] Fu, C. *et al*. Rethinking general underwater object detection: Datasets, challenges, and solutions. *Neurocomputing* **517**, 243–256, 10.1016/j.neucom.2022.10.039 (2023).

[31] Joly, A. *et al*. Lifeclef 2015: Multimedia life species identification challenges. In *Proceedings of the 6th International Conference on Experimental IR Meets Multilinguality, Multimodality, and Interaction (CLEF 2015)*, pages 462-483. Springer, 10.1007/978-3-319-24027-5_46 (2015).

[32] LifeCLEF, *LifeCLEF 2015 Fish Identification Task Dataset*, Available at: https://www.imageclef.org/lifeclef/2015/fish, accessed June 2026.

[33] Joly, A. *et al*. Lifeclef 2017 lab overview: Multimedia species identification challenges. In *Experimental IR Meets Multilinguality, Multimodality, and Interaction*, volume 10456 of *Lecture Notes in Computer Science*. Springer, 10.1007/978-3-319-65813-1_24 (2017).

[34] Fisher, R. B. *et al*. *Fish4Knowledge: Collecting and Analyzing Massive Coral Reef Fish Video Data*, 104. Springer, 10.1007/978-3-319-30208-9 (2016).

[35] Li, W. *et al*. When trackers date fish: A benchmark and framework for underwater multiple fish tracking. In *Proceedings of the Fortieth AAAI Conference on Artificial Intelligence and Thirty-Eighth Conference on Innovative Applications of Artificial Intelligence and Sixteenth Symposium on Educational Advances in Artificial Intelligence*, AAAI Press, 718, 10.1609/aaai.v40i8.37574 (2026).

[36] Panetta, K., Kezebou, L., Oludare, V. & Agaian, S. Comprehensive underwater object tracking benchmark dataset and underwater image enhancement with gan. *IEEE Journal of Oceanic Engineering* **47**(1), 59–75, 10.1109/JOE.2021.3086907 (2022).

[37] Zhang, C. *et al*. Webuot-1m: advancing deep underwater object tracking with a million-scale benchmark, in Advances in Neural Information Processing Systems, vol. 37, A. Globerson, L. Mackey, D. Belgrave, A. Fan, U. Paquet, J. Tomczak, & C. Zhang, Eds. Curran Associates, Inc., pp. 50152– 50167, 10.52202/079017-1587 (2024).

[38] Ravi, N. *et al*. Sam 2: segment anything in images and videos. arXiv preprint arXiv:2408.00714, 10.48550/arXiv.2408.00714 (2024).

[39] Cheng, H. K., Oh, S. W., Price, B., Lee, J. Y. & Schwing, A. Putting the object back into video object segmentation. In *Proceedings of the IEEE/CVF Conference on Computer Vision and Pattern Recognition (CVPR)*, 3151–3161, 10.1109/CVPR52733.2024.00304 (2023).

[40] Catalan, I. A. *et al*. Automatic detection and classification of coastal mediterranean fish from underwater images: Good practices for robust training. *Frontiers in Marine Science* **10**, 1151758, 10.3389/fmars.2023.1151758 (2023).

[41] Sanchez, J. & Lisani, J. Open source video annotation tool for underwater scenes. In *Artificial Intelligence Research and Development* 205-208, 10.3233/FAIA240437 (2024).

[42] Sánchez, J., Lisani, J.-L., Catalán, I. & Alvarez-Ellacuria, A. *SFISHTRACK, Zenodo*10.5281/zenodo.20617668 (2026).

[43] Maggiolino, G., Ahmad, A., Cao, J. & Kitani, K. Deep oc-sort: Multi-pedestrian tracking by adaptive re-identification. In *Proceedings of the IEEE International Conference on Image Processing (ICIP)* 3025–3029, 10.1109/ICIP49359.2023.10222576 (2023).

[44] Bernardin, K. & Stiefelhagen, R. Evaluating multiple object tracking performance: the CLEAR MOT metrics. *EURASIP Journal on Image and Video Processing* **2008**(1), 246309, 10.1155/2008/246309 (2008).

[45] Luiten, J. *et al*. HOTA: A higher order metric for evaluating multi-object tracking. *International Journal of Computer Vision*, **129**(2), 548-578, 10.1007/s11263-020-01375-2 (2021). 10.1007/s11263-020-01375-2 PMC7881978 33642696

[46] Fu, C. *et al*. *RUOD: Real-world Underwater Object Detection Dataset, GitHub repository* https://github.com/xiaoDetection/RUOD accessed June 2026.

[47] Fish4Knowledge Consortium. *Fish4Knowledge Project and Dataset* https://homepages.inf.ed.ac.uk/rbf/Fish4Knowledge/GROUNDTRUTH/ accessed June 2026.

[48] Sánchez, J., Lisani, J.-L., Catalán, I. & Alvarez-Ellacuria, A. *SFISHTRACK: A Dataset for Fish Segmentation and Tracking in Underwater Videos*. *Zenodo*10.5281/zenodo.20617668 (2026).10.1038/s41597-026-07786-z42586979

